# Feasibility of equivalent performance of 3D TOF [^18^F]-FDG PET/CT with reduced acquisition time using clinical and semiquantitative parameters

**DOI:** 10.1186/s13550-021-00784-9

**Published:** 2021-05-01

**Authors:** Julia Pilz, Lukas Hehenwarter, Georg Zimmermann, Gundula Rendl, Gregor Schweighofer-Zwink, Mohsen Beheshti, Christian Pirich

**Affiliations:** 1 Department of Nuclear Medicine and Endocrinology, University Hospital Salzburg, Paracelsus Medical University, Salzburg, Austria; 2 Team Biostatistics and Big Medical Data, IDA Lab Salzburg, Paracelsus Medical University, Salzburg, Austria

**Keywords:** [^18^F]-FDG PET/CT, Short acquisition time, Diagnostic equivalence, 3D Time-of-flight

## Abstract

**Background:**

High-performance time-of-flight (TOF) positron emission tomography (PET) systems have the capability for rapid data acquisition while preserving diagnostic image quality. However, determining a reliable and clinically applicable cut-off of the acquisition time plays an important role in routine practice. This study aimed to assess the diagnostic equivalence of short acquisition time of 57 with routine 75 seconds per bed position (s/BP) of [^18^F]-fluoro-deoxy-glucose (FDG) PET.

Phantom studies applying EARL criteria suggested the feasibility of shortened acquisition time in routine clinical imaging by 3D TOF PET/CT scanners. Ninety-six patients with melanoma, lung or head and neck cancer underwent a standard whole-body, skull base-to-thigh or vertex-to-thigh [^18^F]-FDG PET/CT examination using the 3D TOF Ingenuity TF PET/CT system (Philips, Cleveland, OH). The [^18^F]-FDG activity applied was equal to 4MBq per kg body weight. Retrospectively, PET list-mode data were used to calculate a second PET study per patient with a reduced acquisition time of 57 s instead of routine 75 s/BP. PET/CT data were reconstructed using a 3D OSEM TOF algorithm. Blinded patient data were analysed by two nuclear medicine physicians. The number of [^18^F]-FDG-avid lesions per body region (head&neck, thorax, abdomen, bone, extremity) and image quality (grade 1–5) were evaluated. Semiquantitative analyses were performed by standardized uptake value (SUV) measurements using 3D volume of interests (VOI). The visual and semiquantitative diagnostic equivalence of 214 [^18^F]-FDG-avid lesions were analysed in the routine standard (75 s/BP) as well as the calculated PET/CT studies with short acquisition time. Statistical analyses were performed by equivalence testing and Bland–Altman plots.

**Results:**

Lesion detection rate per patient’s body region agreed in > 98% comparing 57 s/BP and 75 s/BP datasets. Overall image quality was determined as equal or superior to 75 s in 80% and 69%, respectively. In the semiquantitative lesion-based analyses, a significant equivalence was found between the 75 s/BP and 57 s/BP PET/CT images both for SUV_max_ (*p* = 0.004) and SUV_mean_ (*p* = 0.003).

**Conclusion:**

The results of this study demonstrate significant clinical and semiquantitative equivalence between short acquisition time of 57 s/BP and standard 75 s/BP 3D TOF [^18^F]-FDG PET/CT scanning, which may improve the patient’s workflow in routine practice.

## Introduction

Positron emission tomography/computed tomography (PET/CT) has been widely implemented as a diagnostic tool in the field of oncology, cardiology and neurology in clinical routine [1–4]. The most commonly employed PET radiopharmaceutical is [^18^F]-fluoro-deoxy-glucose (FDG), a glucose analogue [2, 5, 6].

In 2015, the European Association of Nuclear Medicine (EANM) published the current guidelines for the calculation of [^18^F]-FDG activity to be applied based on the patient’s body weight (bw), scanner type and PET acquisition time [2]. However, current EANM guidelines defining PET acquisition time and [^18^F]-FDG activity calculation may not exactly reflect the ongoing technical improvements in PET/CT imaging [3, 7–10], which allow reductions in PET acquisition time or [^18^F]-FDG activity applied while keeping high image quality [7, 11–13]. One of the major technical advancements in PET/CT scanners was the implementation of the time-of-flight approach leading to better performance of this modality and more accurate localization of the annihilation process [14]. Hence, image reconstruction and image quality could be substantially improved [15].

These advances could enable further reduction of [^18^F]-FDG activity applied, which is highly desirable in clinical routine keeping the radiation exposure for patients and hospital staff as low as reasonably achievable (ALARA principle) [12, 16].

In addition, the possibility to reduce the PET acquisition time per bed position will improve the workflow of the PET/CT centres as well as the patient comfort [15]. Based on our previous phantom study results, the shortest EARL approved and equivalent time of acquisition was equal to 57 s per bed position [17]. In this study, we have tried to assess the clinical performance of the short 57 s/BP PET acquisition time with validated standard PET acquisition time of 75 s/BP. Furthermore, semiquantitative data were compared between short and standard PET acquisitions using “equivalence testing”, as a novel approach.

## Material and methods

### Patient population

The [^18^F]-FDG PET/CT data of 96 consecutive patients with histopathologically verified melanoma, lung or head and neck cancer were retrospectively analysed. These three cancers types were selected for the following reasons: Firstly, those are highly referred cancer patients for PET/CT imaging in our centre, secondly, [^18^F]-FDG-PET/CT studies are routinely integrated into the work-up of these cancer types and thirdly, there exists an integrated common and standardized histopathologic work-up of [^18^F]-FDG-avid lesions. Patients were divided into three different weight classes (< 75 kg, 75–100 kg, > 100 kg). Patient demographics are shown in Table 1. Exclusion criteria were pregnancy, age < 18 years and a glucose level > 195 mg/dl measured before tracer application. This research was conducted according to the principles of the Declaration of Helsinki and all subsequent revisions and was approved by the routing Ethics Committee of the province (EC-number: 415-E/2491/2-2019). All data were carefully anonymized to fulfil the regulations regarding data protection.

**Table 1:** Demographics of study population

Patient #	Disease	Male	Female	Mean age (y) ± SD	< 75 kg bw	75–100 kg bw	> 100 kg bw	Mean applied MBq ± SD
33	Melanoma	18	15	65.9 ± 13.5	11	12	10	341.6 ± 69.3
34	Lung cancer	21	13	65.8 ± 8.3	10	10	14	351.9 ± 73.8
29	Head & neck cancer	21	8	63.3 ± 12.6	10	11	8	342.0 ± 69.5

### PET/CT examination and data reconstruction

All 6-h fasting cancer patients underwent a standard whole-body (melanoma), vertex-to-thigh (head and neck) or skull base-to-thigh (lung) [^18^F]-FDG PET/CT examination using our EARL accredited 3D TOF Ingenuity TF PET/CT system (Philips, Cleveland, OH) including a low-dose CT with 100 kV and 45 mAs for anatomical localization and attenuation correction purposes. Each patient received 4 MBq per kg bw (range 246–479 MBq; mean ± SD 345 ± 71 MBq) of [^18^F]-FDG. The mean uptake time was 60 min ± 12 min as recommended by the European Association of Nuclear Medicine Research Ltd (EARL) [2]. All patients had to empty their bladder before PET/CT scanning. Following low-dose CT scan, PET acquisition was performed with a duration of 75 s per bed position and a bed overlap of 50% over the same anatomical area. PET data were reconstructed using the vendor-recommended blob-based ordered-subset expectation maximization (OSEM) time of flight (TOF) algorithm [18] with the default setting of 3 iterations and 33 subsets and a matrix of 144 × 144 with a voxel size of 4 × 4 × 4 mm^3^.

Transverse and axial spatial resolution of our PET scanner was equal to 4.7 mm, the TOF system sensitivity in the centre was > 18830 cps/MBq and the timing resolution of TOF performance was equal to 495 ps.

No post-reconstruction smoothing filter was used. All image data received correction for random coincidences, normalization, dead time losses, scatter and attenuation as recommended by the EANM guidelines version 1.0 [5].

Additionally, a second imaging dataset per patient with a short acquisition time of 57 s per bed position was reconstructed using PET list-mode data. All image reconstruction settings were identical.

### Imaging analysis

The data of 192 anonymized PET/CT studies, two datasets per patient (75 s and 57 s PET acquisition time), were analysed using the Intellispace software version 10.1 (Philips Medical Systems, The Netherlands) by two experienced board-certified nuclear medicine physicians blinded to any information concerning patient, indication, PET acquisition time or applied radiotracer activity. [^18^F]-FDG-avid lesions per body region (head & neck, thorax, abdomen, bone, extremity) and image quality (grade 1-5), further demonstrated in Table 2, were evaluated. Criteria for image quality receiving grade ≥ 3 were image reconstruction artefacts, noisy images, irregular shape or contour of lesions and a low lesion to background ratio. Reader 1 and Reader 2 categorized the [^18^F]-FDG-avid lesions per body regions in three groups, 0, 1–4 and > 4 lesions in order to generate reports for all 192 PET/CT datasets. After the completion of the reading, the report results with the categorized lesions were compared with the standard and recalculated PET/CT studies with reduced acquisition time.

SUV_max_ and SUV_mean_ were calculated with the help of 3D VOI for 214 [^18^F]-FDG-avid lesions matched per patient between the 75 s and 57 s PET/CT studies. The matched lesions were mainly defined based on their anatomical localization and their morphological pattern on CT. For each matched lesion, the SUV was determined using the same threshold- and volume-based VOI determination in order to compare the SUV as precisely as possible. Firstly, we defined a 40% threshold for drawing an automatic VOI over the abnormal matched lesions. Secondly, we tried to adjust this threshold manually to receive the same volume on both matched lesions and therefore avoid any possible influence of different VOIs on the results.

As a measure for image noise, SUV_mean_ plus standard deviation were additionally measured by drawing a circular region of interest (ROI) in normal liver tissue and the mediastinum in order to calculate the coefficient of variation (COV). The diameter of the ROIs was equal to 7.4 ± 0.9 cm (liver) and 4.3 ± 0.8 cm (mediastinum). The COV, which is defined as the standard deviation divided by the SUV_mean_ [19], was evaluated for liver and mediastinum region for each PET/CT dataset.

**Table 2:** Description of different grades for the assessment of image quality

Grade 1	Excellent
Grade 2	Good
Grade 3	Average
Grade 4	Poor but interpretable
Grade 5	Poor not interpretable

### Statistical analysis

A comparison of the mean of differences of SUV_max_ and SUV_mean_ of the 75 s and 57 s PET/CT datasets was performed by equivalence testing (TOST) [20] using the R software version 3.6.3 [21]. Due to the lack of objective and clinically sensitive equivalence margins on the original scale of SUV_max_ and SUV_mean_, standardized equivalence margins (i.e. Cohen’s d) equal to 0.2 were used. This means that only very small SUV differences between 75 s and 57 s PET/CT studies would be tolerated. For all equivalence tests, the alpha level was set to 0.05.

Descriptive statistics of continuous data were represented by the calculation of the mean ± SD, minimum and maximum. Frequencies in percent were evaluated for categorical variables, e.g. image quality.

Bland–Altman plots, the method of choice for interpreting comparison studies [22], were created with GraphPad Prism 6 for assessing the agreement of SUV_max_ and SUV_mean_ between the 75 s and 57 s PET/CT datasets.

We would like to mention that from a subject-matter point of view, it might seem counterintuitive to use equivalence tests instead of non-inferiority tests. However, we prefer the former approach, because it aligns well with the two-sided concept of the visual inspections (i.e. the Bland–Altman plots). From a statistical point of view, both tests are equivalent in our setting anyway, because the superiority case is highly unlikely if not impossible at all, and therefore, the *p* values of the TOST and the non-inferiority tests will be the same.

## Results

The imaging data of 96 oncological patients (60 men, 36 women, age 27–87, mean ± SD 65 ± 11.6 years) were analysed in this retrospective study.

### Visual analyses

Equal report results were found in > 98% of the 480 investigated body regions (5 body regions per patient: head & neck, thorax, abdomen, bone, extremity) for both readers comparing 75 and 57 s PET/CT datasets. Of 480 analysed patient’s body regions, 9 (1.88%) were categorized differently concerning the number of [^18^F]-FDG-positive lesions by reader 1, whereas reader 2 investigated 5 (1.04%) differently (see Table 3). Table 4 summarizes, for both investigators, the results of lesion classification per patient’s body region. An additional visual analyses of the images showed that all matched PET/CT datasets, 75 s and the corresponding 57 s per patient, showed the same number of [^18^F]-FDG-positive lesions.

**Table 3:** Blinded rating of 96 PET datasets per acquisition time

	Total number of investigations	Investigated body regions rated equally	Investigated body regions rated differently
Reader 1	480	471	9
Reader 2	480	475	5

Each patient’s investigation is divided into 5 different body regions leading to 480 body regions to investigate per acquisition time

**Table 4:** Analysis of 480 body-region classifications: reader 1 and reader 2

	0 lesions	1–4 lesions	> 4 lesions
*Reader 1*			
Head & Neck	69	24	3
Thorax	62	21	13
Abdomen	86	6	4
Bone	85	7	4
Extremity	92	3	1
*Reader 2*			
Head & Neck	76	18	2
Thorax	64	20	12
Abdomen	87	6	3
Bone	86	8	2
Extremity	94	1	1

The image quality of short acquisition was assessed as equal in 62.5% and superior in 17.7% by reader 1. Moreover, reader 2 rated the image quality of short acquisition as even in 42.7% and superior in 26%. Therefore, the overall subjective image quality was described as equal or superior in 80.2 and 68.7%, respectively. It is noteworthy that no PET/CT dataset with short acquisition time was graded as not interpretable. All image quality grades of both readers are provided in Table 5.

**Fig. 1:**
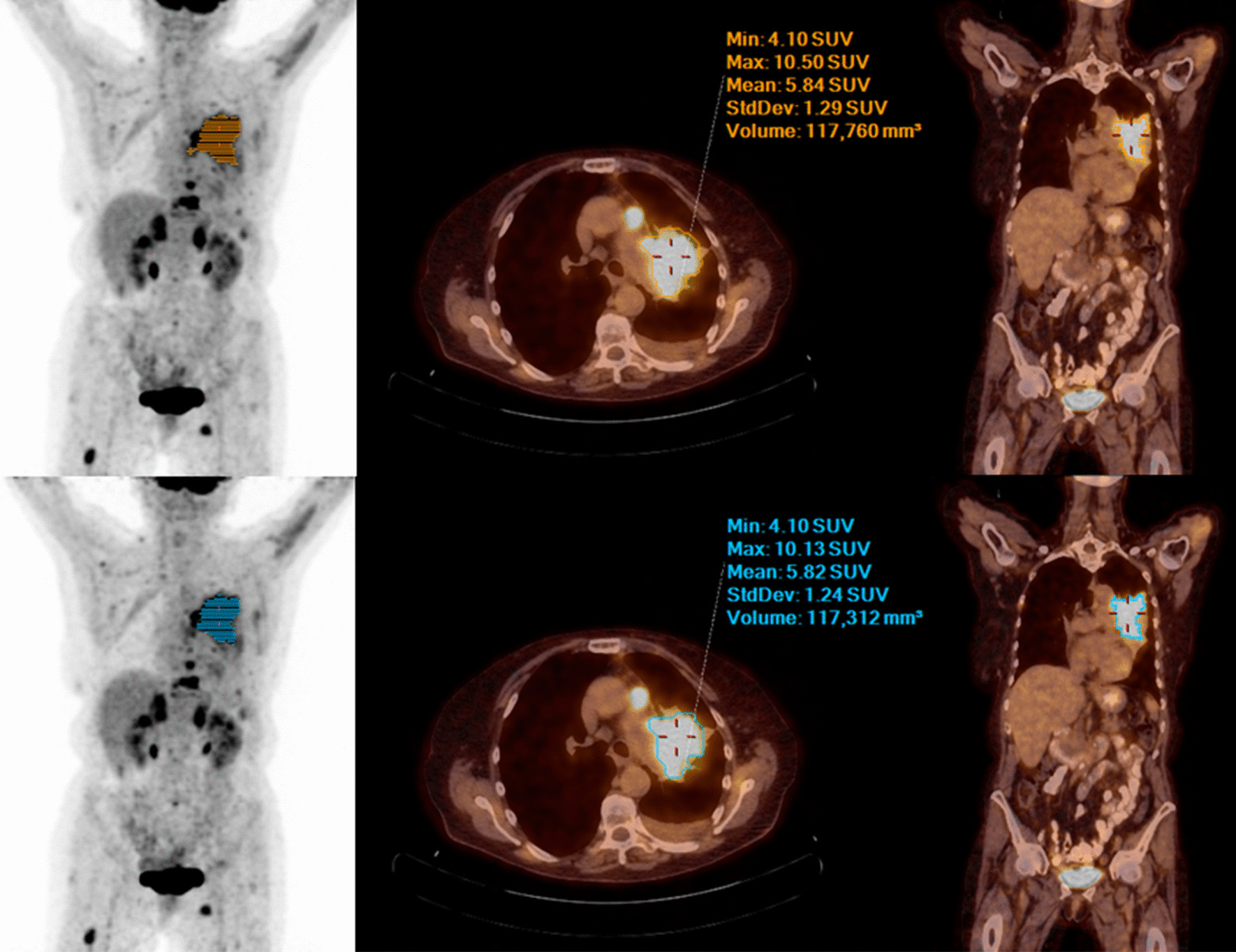
MIP, fused axial and coronal PET/CT images (from left to right) with 75 s (upper row )and 57 s (lower row) PET acquisition time per bed position of a 71-year-old female patient with 65 kg bw suffering from lung cancer—no significant difference in image quality, lesion detectability and quantification

**Fig. 2:**
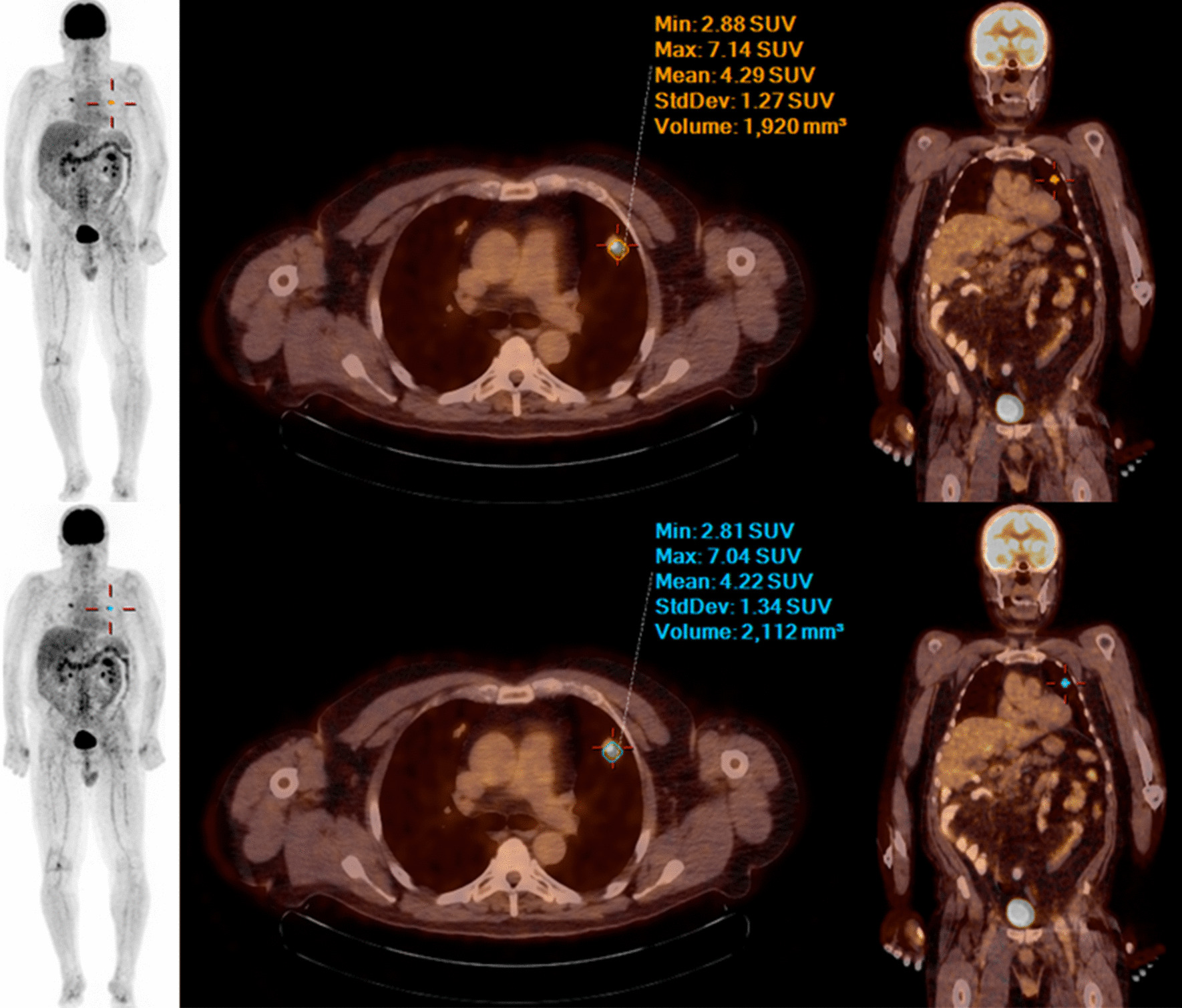
MIP, fused axial and coronal PET/CT images (from left to right) with 75 s (upper row )and 57 s (lower row) PET acquisition time per bed position of a 63-year-old male patient with 94 kg bw suffering from melanoma—no significant difference in image quality, lesion detectability and quantification

**Fig. 3:**
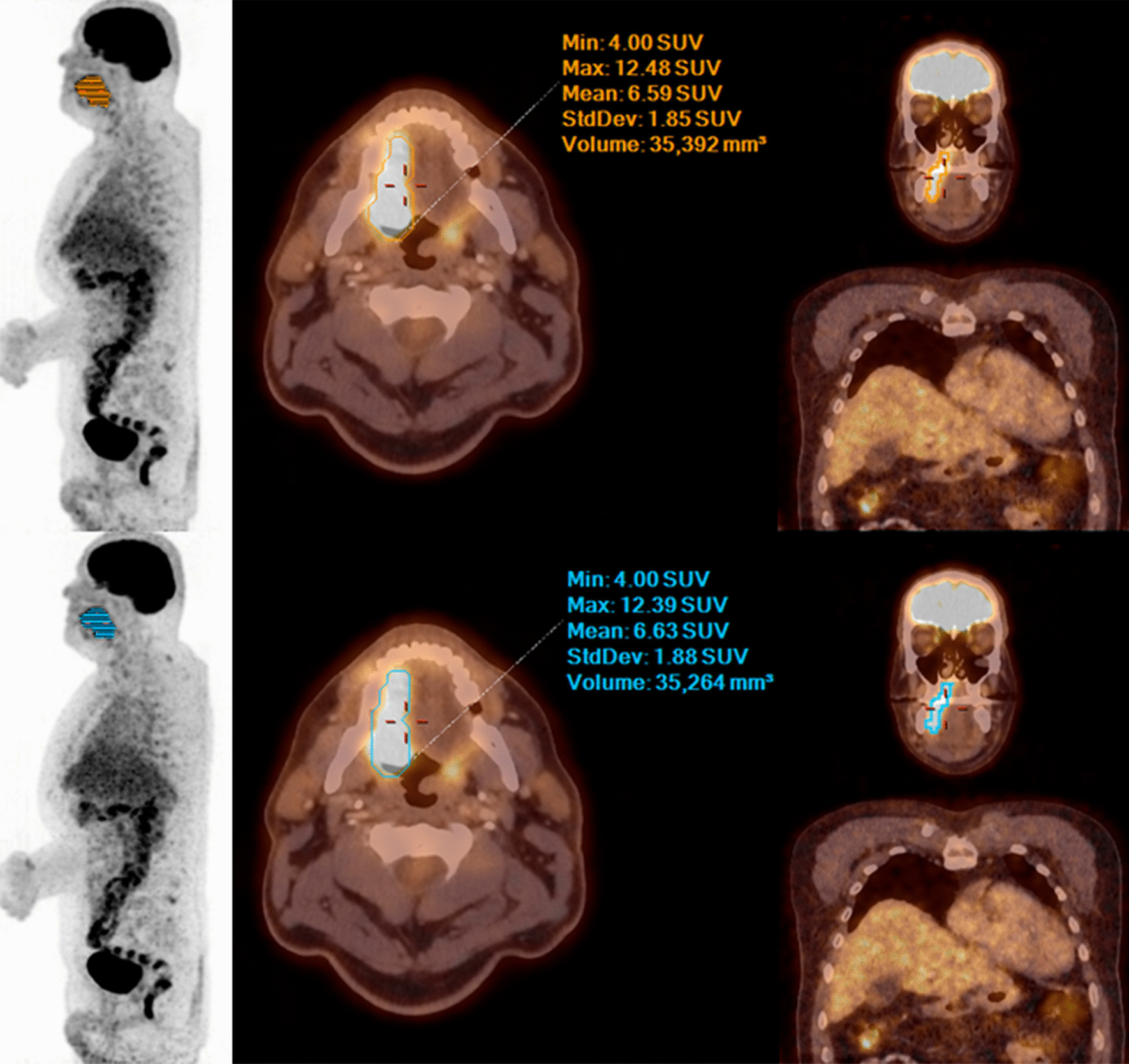
MIP, fused axial and coronal PET/CT images (from left to right) with 75 s (upper row )and 57 s (lower row) PET acquisition time per bed position of a 57-year-old male patient with 105 kg bw suffering from head & neck cancer—no significant difference in image quality, lesion detectability and quantification

Figures 1, 2 and 3 show examples of PET/CT images of patients suffering from melanoma, lung or head and neck cancer with < 75 kg, 75–100 kg and > 100 kg.

**Table 5:** Two crosstabs of image quality: grading of the 75 s and 57 s PET dataset -  reader 1 and reader 2

	Grade 1	Grade 2	Grade 3	Grade 4
	(75 s)	(75 s)	(75 s)	(75 s)
*Reader 1*				
Grade 1 (57 s)	29	13	0	0
Grade 2 (57 s)	15	29	4	0
Grade 3 (57 s)	0	4	2	0
Grade 4 (57 s)	0	0	0	0
*Reader 2*				
Grade 1 (57 s)	17	22	1	0
Grade 2 (57 s)	26	22	1	1
Grade 3 (57 s)	0	3	2	0
Grade 4 (57 s)	0	0	1	0

### Semiquantitative analyses

**Fig. 4:**
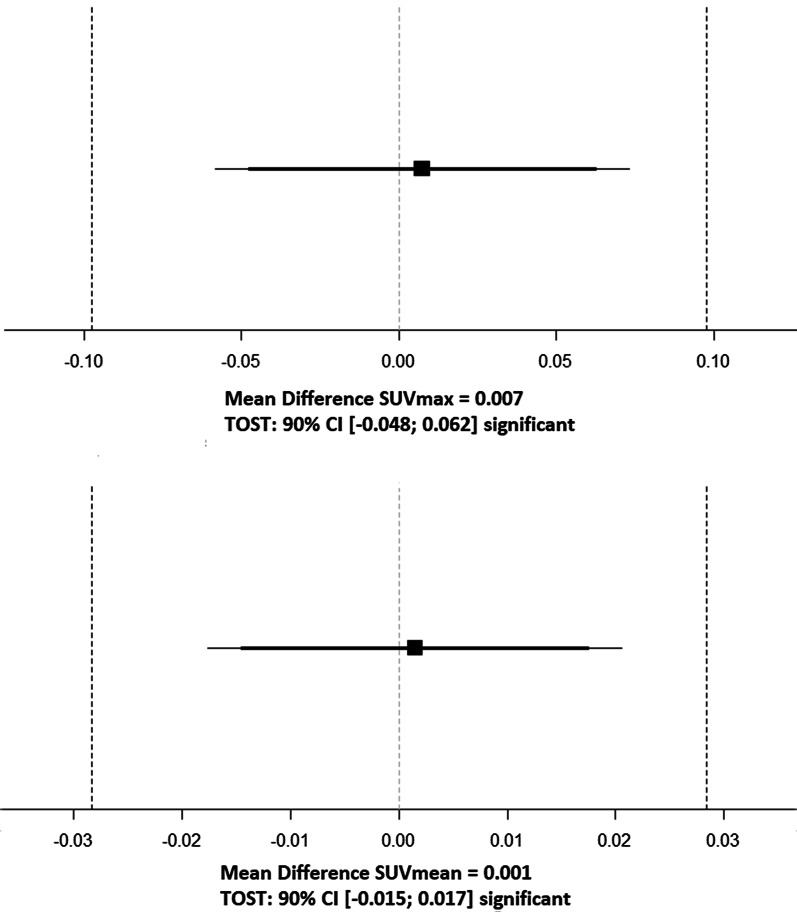
Comparison of SUV_max_ 75 s and 57 s PET/CT datasets and SUV_mean_ 75 s and 57 s PET/CT images of 214 [^18^F]-FDG-avid lesions by performing the TOST equivalence test [20]

SUV_max_ and SUV_mean_ were calculated for 214 [^18^F]-FDG-avid lesions (164 malignant: mean SUV_max_ 7.9 ± 4.0, mean SUV_mean_ 4.8 ± 1.9, 50 benign: mean SUV_max_ 6.0 ± 3.3, mean SUV_mean_ 4 ± 1.4) matched per patient between the 75 s and 57 s [^18^F]-FDG PET/CT studies. Mean difference of SUV_max_ ± SD was equal to 0.0074 ± 0.49, and the 90% confidence interval (CI) ranged from − 0.048 to − 0.062. Moreover, the mean difference of SUV_mean_ ± SD was equal to 0.0015 ± 0.14. The 90% CI ranged from − 0.015 to − 0.017. Since the CIs lay within the equivalence bounds, the equivalence tests were significant. Accordingly, the obtained *p* values were 0.004 for SUV_max_ and 0.003 for SUV_mean_. An illustrative visualization for the equivalence tests is visualized in Fig. 4.

**Fig. 5:**
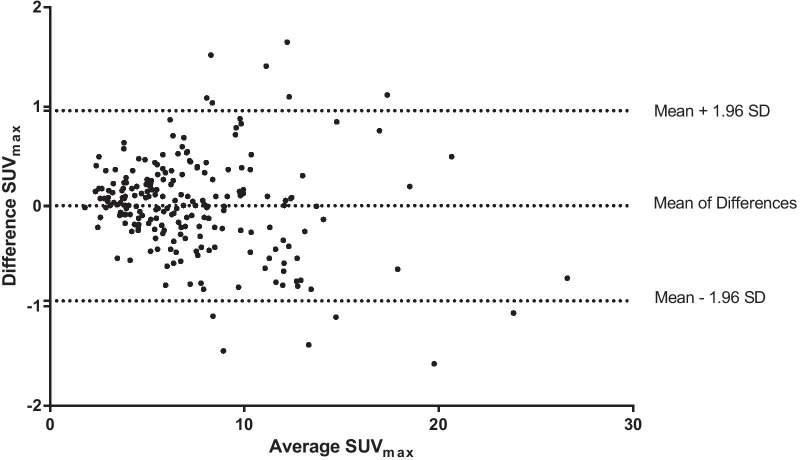
Bland–Altman plot showing SUV_max_ measurement differences of 214 lesions between the 75 s and 57 s PET/CT datasets plotted against the mean SUV_max_. In order to interpret these powerful Bland–Altman plots properly, it is essential to know that the differences between the quantified SUVs are plotted on the *y-*axis and the average of the SUVs is visualized on the *x-*axis

**Fig. 6:**
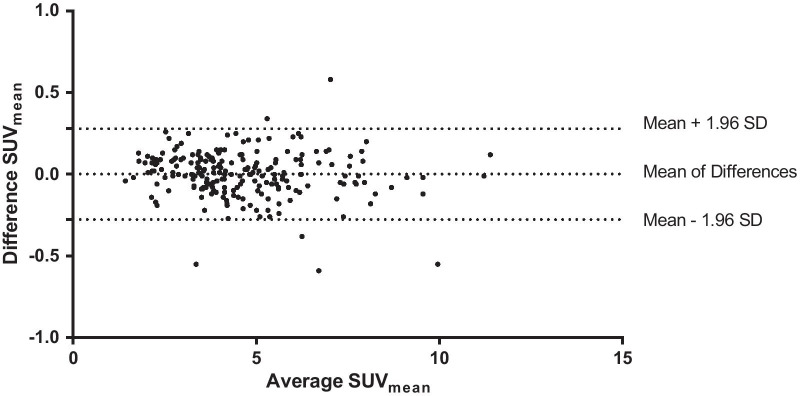
Bland–Altman plot showing SUV_mean_ measurement differences of 214 lesions between 75 s and 57 s PET/CT datasets plotted against the mean SUV_mean_. In order to interpret these powerful Bland–Altman plots properly, it is essential to know that the differences between the quantified SUVs are plotted on the *y-*axis, and the average of the SUVs is visualized on the *x-*axis

Bland–Altman plots show good agreement between the SUVs of the 75 s and 57 s [^18^F]-FDG PET/CT datasets (see Figs. 5 and 6). Furthermore, no systematic bias was observed in the quantification of SUV_max_ and SUV_mean_.

### Image noise

**Fig. 7:**
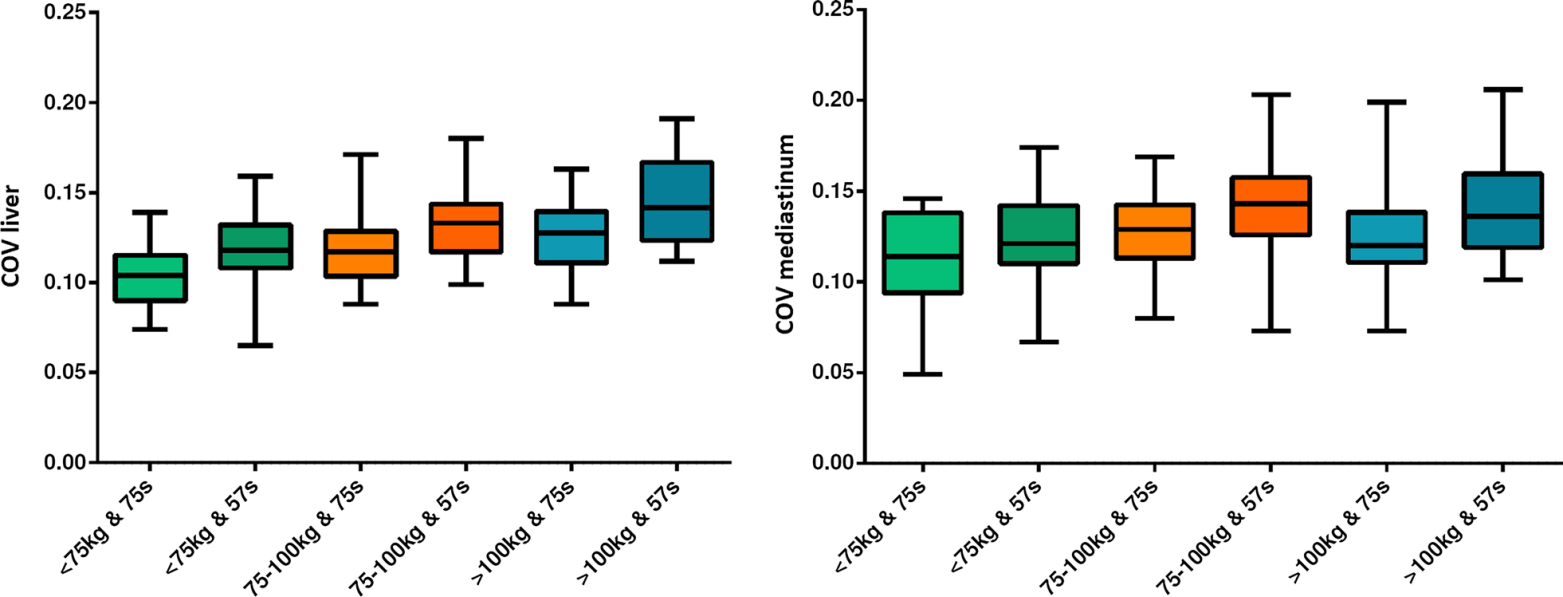
Boxplots representing COV_liver_ and COV_mediastinum_ for different bw classes - 75 s and 57 s PET/CT acquisition time

The overall COV_liver_ was equal to 0.12 ± 0.02 for 75 s PET/CT datasets and 0.13 ± 0.02 for the 57 s PET/CT datasets, while the COV_mediastinum_ resulted in 0.12 ± 0.02 for the 75 s PET/CT datasets and 0.14 ± 0.03 for the 57 s PET/CT datasets. As expected, the COV was increasing for patients with high body weight. Details of the calculated COVs for patient groups with different body weights are demonstrated in Fig. 7.

## Discussion

Since experimental [19, 23] and clinical [23–25] evidence suggested the feasibility to shorten PET acquisition times beyond EANM recommendations, we explored the effect of PET scan time reduction on quantitative segmentation parameters, lesion detectability and image quality using the Ingenuity TF PET/CT system.

Our retrospective patient study, including 96 patients with very common cancers, e.g. melanoma, lung or head and neck cancer, demonstrates diagnostic equivalence of PET imaging datasets with 75 s and 57 s acquisition time.

In contrast to other studies dealing with PET scan time reduction, this paper is novel since the comparison of quantitative segmentation parameters between the standard and reduced PET acquisition times are validated by equivalence testing. Our findings seem very plausible due to improvements in PET technology and image reconstruction, e.g. TOF [7], implemented in PET/CT scanners used in clinical routine. TOF technology allows a more accurate localization of the annihilation process leading to increased contrast and reduced noise levels of PET/CT studies [7]. These advantages allow shorter PET acquisition times [7], e.g. 57 s per bed position, while still maintaining good image quality and diagnostic accuracy. Another strength of our study is the inclusion of patients over a wide range of body weight (40–145 kg) demonstrating the diagnostic equivalence of the 57 s and 75 s acquisitions throughout much of the range of clinically presenting body weight.

Previous studies aiming to optimize PET acquisition time or [^18^F]-FDG activity applied often focused on the calculation of noise equivalent count rate (NECR) [24, 26–29], which is a measure for image signal-to-noise ratio of PET/CT scanners. However, clinical experience suggests that quantitative measurements (e.g. SUV) of imaging performance are more appropriate guides for the assessment of lesion detectability and PET image quality [30]. Comparable studies [15, 23, 25] (see Table 6) either included less PET patient data, evaluated different PET/CT scanners, used other methods for statistical analyses or demonstrated a higher cut-off for PET acquisition time.

A similar study from Halpern et al. stated that a PET acquisition time of 60 s per bed position is only sufficient for patients with a body weight < than 59kg, although they injected 7.7 MBq/kg/bw [25]. However, the PET/CT scanner described in this paper, which has already been published in 2004, did not have TOF technology. Our study specifically included 32 patients weighing more than 100 kg, and the activity applied was equal to 4 MBq/kg/bw. Figure 7 shows that there is no significant COV difference for PET datasets of patients with a bw > 100 kg between the 75 s and 57 s acquisition times.

On the other hand, Murray et al. demonstrated that even with 15-s acquisition time per bed position [^18^F]-FDG-avid lesions larger than 2 cm were visible and 2D calculated SUV values were comparable. However, this study compared non-TOF with TOF image reconstruction, and for the statistical analysis no equivalence test was performed [23]. The SUV calculation of our 214 [^18^F]-FDG-avid lesions is based on 3D volumetric data (range 2.1–253312 mm^3^ = 0.002 ml−253.312 ml) used also for equivalence testing. We would like to emphasize that equivalence testing is considered as the state of the art and statistically reliable method for demonstrating equality of two investigated methods [20]. Furthermore, our lesion detection rate per patient’s body region matched in > 98% for both investigators comparing 75 and 57 s PET/CT datasets. All [^18^F]-FDG-positive lesions were visible in the 75 s as well as the corresponding 57 s PET/CT dataset, and the image quality of short acquisition datasets was rated equal or superior in 80 and 69%.

Another study with the aim to evaluate the scanner performance of a digital PET/CT system concluded a minimal PET acquisition time of 90s/BP [15]. However, this newest generation scanner shows higher sensitivity, better spatial resolution and highly improved TOF resolution compared to our Ingenuity TF PET/CT system. It is important to mention though that the mean [^18^F]-FDG uptake time was equal to 101 min. This increased delay was caused by a performance of an analog PET/CT examination beforehand.[15]

The results of this study are promising when applying a short PET acquisition time of 57 s/BP with comparable diagnostic accuracy and adequate image quality to standard acquisition time. This is highly beneficial since a reduced PET scan time improves patient comfort, which is especially important for many elder or anguished patients, who are more likely to move during the imaging procedure [25]. Accelerated PET imaging can avoid motion artefacts in the latter patient population and increase patient throughput, which meets the ever-increasing demand for PET studies [1] due to an increasing number of well-evidenced clinical indications.

Further benefits can be experienced in the paediatric field where PET/CT studies with short acquisition times might lead to a decrease in anaesthesia or sedation time [15, 31].

Certain limitations of our study should be taken into account. As already mentioned, the PET acquisition time reduction was investigated by a retrospectively designed patient study. PET/CT datasets with 57 s acquisition time per bed position were calculated using PET-listmode data. Further prospective investigations are necessary for defining the lowest cut-off of PET acquisition time, especially when reducing tracer activity. Furthermore, this study protocol was planned for the Ingenuity TF PET/CT scanner. Although we believe that the described methodology of this study can be easily translated to other 3D TOF PET/CT scanner, these results may not be generalized for all PET/CT scanners from different vendors.

Since the focus of this study was to assess the impact of the PET results on routine clinical practice, reader 1 and reader 2 were asked to perform a global disease assessment (0, 1-4 or > 4 lesions per body region), and therefore, no counting of exact lesion numbers was required for their blind readings.

**Table 6:** Comparison of studies focusing on PET acquisition time

Author	Study type	[^18^F]-FDG activity	PET/CT scanner & reconstruction	PET acquisition time	Findings
Halpern et al. [25]	patient (*n* = 57)	7.77 MBq/kg/bw	Reveal RT PET/CT + 3D OSEM	120s, 60s	60s/BP only for patients < 59 kg
Murray et al. [23]	phantom + patient (*n* = 20)	350 ± 40 MBq	Gemini TF PET/CT + 3D-RAMLA & OSEM TOF	60s, 30s, 15s, 10s	15s/BP: approved lesion detectability
Sonni et al. [15]	patient (*n* = 58)	356 ± 37 MBq	Discovery MI PET/CT + 3D PSF TOF	120s, 90s, 60s, 30s	90s/BP with good image quality
Pilz et al.	patient (*n* = 96)	4 MBq/kg/bw	Ingenuity TF PET/CT + 3D OSEM TOF	75s, 57s	57 s/BP = Approved acquisition time

## Conclusion

This retrospective study of clinical PET/CT data successfully demonstrates the diagnostic equivalence of [^18^F]-FDG PET/CT studies using short acquisition time of 57 s per bed position showing equivalent lesion detectability and adequate image quality comparing to standard PET acquisition time.

## Data Availability

The datasets used and analysed during this study are available from the corresponding author on reasonable request.

## Abbreviations

ALARA: As low as reasonably achievable
Bw: Body weight
CI: Confidence interval
COV: Coefficient of variation
EANM: European Association of Nuclear Medicine
EARL: EANM Research Ltd
[^18^F]-FDG: Fluor-18 fluoro-deoxy-glucose
NECR: Noise equivalent count rate
OSEM: Ordered-subset expectation maximization
PET/CT: Positron emission tomography/computed tomography
PSF: Point spread function
ROI: Region of interest
s/BP: Seconds per bed position
SD: Standard deviation
SUV: Standardized uptake value
TOF: Time-of-flight
VOI: Volume of interest
